# Ultrasound-mediated cavitation does not decrease the activity of small molecule, antibody or viral-based medicines

**DOI:** 10.2147/IJN.S141557

**Published:** 2018-01-10

**Authors:** Rachel Myers, Megan Grundy, Cliff Rowe, Christian M Coviello, Luca Bau, Philippe Erbs, Johann Foloppe, Jean-Marc Balloul, Colin Story, Constantin C Coussios, Robert Carlisle

**Affiliations:** 1 OxSonics Ltd, The Magdalen Centre; 2 BUBBL, IBME, Department of Engineering Science, University of Oxford, Oxford, UK; 3 Transgene SA, Illkirch-Graffenstaden, France

**Keywords:** nanomedicine, antibody, virus, ultrasound, cavitation

## Abstract

The treatment of cancer using nanomedicines is limited by the poor penetration of these potentially powerful agents into and throughout solid tumors. Externally controlled mechanical stimuli, such as the generation of cavitation-induced microstreaming using ultrasound (US), can provide a means of improving nanomedicine delivery. Notably, it has been demonstrated that by focusing, monitoring and controlling the US exposure, delivery can be achieved without damage to surrounding tissue or vasculature. However, there is a risk that such stimuli may disrupt the structure and thereby diminish the activity of the delivered drugs, especially complex antibody and viral-based nanomedicines. In this study, we characterize the impact of cavitation on four different agents, doxorubicin (Dox), cetuximab, adenovirus (Ad) and vaccinia virus (VV), representing a scale of sophistication from a simple small-molecule drug to complex biological agents. To achieve tight regulation of the level and duration of cavitation exposure, a “cavitation test rig” was designed and built. The activity of each agent was assessed with and without exposure to a defined cavitation regime which has previously been shown to provide effective and safe delivery of agents to tumors in preclinical studies. The fluorescence profile of Dox remained unchanged after exposure to cavitation, and the efficacy of this drug in killing a cancer cell line remained the same. Similarly, the ability of cetuximab to bind its epidermal growth factor receptor target was not diminished following exposure to cavitation. The encoding of the reporter gene luciferase within the Ad and VV constructs tested here allowed the infectivity of these viruses to be easily quantified. Exposure to cavitation did not impact on the activity of either virus. These data provide compelling evidence that the US parameters used to safely and successfully delivery nanomedicines to tumors in preclinical models do not detrimentally impact on the structure or activity of these nanomedicines.

## Introduction

There has been much recent controversy surrounding the true potential impact of the enhanced permeability and retention (EPR) effect.^1^ Regardless of the scale of the EPR effect, it is clear that one of the biggest challenges to successful cancer therapy is achieving effective delivery of nanomedicines into and throughout solid tumors.^2^ When gas nuclei in solution are exposed to the alternating rarefactional and compressional cycles of an ultrasound (US) wave, they expand and collapse thereby establishing microstreaming events which can propel substances dissolved in the solution over millimeter distances.^3^ This phenomenon is termed US-mediated cavitation and has been used as a mechanism to enhance the tumoral delivery of a range of oncological therapies.^4^ Notably, this approach can be achieved noninvasively, using relatively low-cost equipment, and even permits real-time feedback on the success of the procedure,^5^ factors which may ultimately provide enormous clinical benefit.

In vitro and preclinical studies to validate US- and/or cavitation-mediated delivery have shown substantial improvements to the accumulation and distribution of small molecule,^6^,^7^ antibody,^8^ nanoparticle^9^,^10^ and gene- and viral-based medicines.^11^,^12^ Indeed, an optimized system which can achieve targeted, sustained cavitation was recently shown to provide up to 10,000-fold enhancement in the tumor infection achieved by an oncolytic vaccinia virus (VV).^13^ Crucially, the development of systems to target, control and monitor the levels of cavitation in real time^5^ has provided a means of ensuring delivery can be achieved without direct damage to cells of the target tissue or vasculature. While these and many other studies^14^ provide confidence in the principle and safety of cavitation-mediated delivery, effective translation into the clinic requires more detailed analysis of the impact of cavitation events on the structure and activity of the therapeutic agents being delivered. In particular, the high levels of microstreaming and shockwave generation associated with cavitation have the potential to break covalent bonds and denature secondary, tertiary and quaternary protein structure.^15^ Such potential is especially pertinent to the most sophisticated and promising new anticancer agents, which are increasingly biological-based nanomedicines.^16^ Here, we established a highly controlled environment in which we then tested the impact of cavitation on a small molecule (doxorubicin, Dox), an antibody (cetuximab) and non-enveloped (adenovirus, Ad) and enveloped (VV) viral vectors. We demonstrate that exposure to the same levels and durations of cavitation that have been shown to enhance delivery in vitro and in preclinical murine models^13^ does not detrimentally impact on the activity of agents representative of small molecule, antibody and virus-based drugs.

## Materials and methods

### Sonosensitive particles and drugs

Cup-shaped gas-stabilizing sonosensitive particles (SSPs)^8^ provided the nuclei for initiation of sustained controlled cavitation and were prepared at a concentration of 25 mg/mL and sterilized by gamma sterilization. They were then mixed with 3.5 mL fetal bovine solution (FBS; Fisher, 11550356) in a cuvette to have a final concentration of 1.27 mg/mL. To characterize the impact of cavitation on different therapeutics, Dox (Sigma, D1515), cetuximab (obtained from Oxford University Hospitals NHS Foundation Trust, UK), VV (both luciferase-expressing and non-expressing versions [TG6002], Transgene SA, France) and Ad (Native Antigen Company, Oxford, UK) were mixed with the SSP suspension to have final concentrations of 455 μM, 126.9 μg/mL, 5.4×10^4^ plaque-forming units/mL and 3.1×10^9^ viral particles/mL, respectively. These concentrations and conditions aimed to mimic those experienced in preclinical studies as closely as possible.

### Cavitation test rig

**Figure 1:**
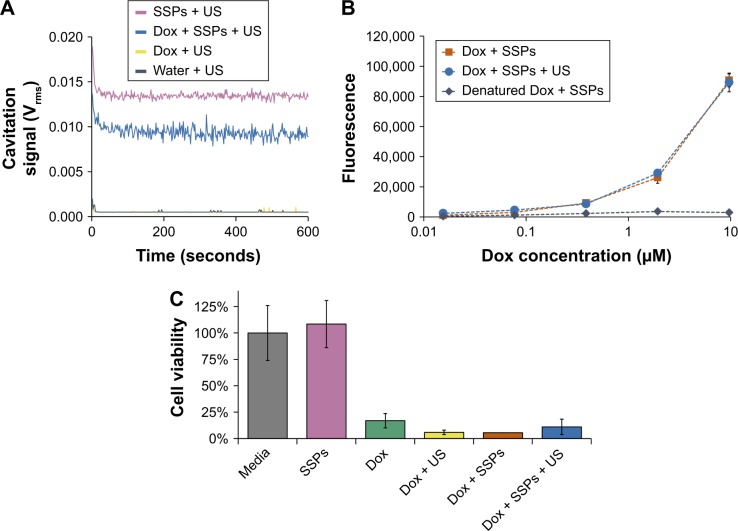
Impact of cavitation on the fluorescence and cytotoxicity of Dox. **Notes:** (**A**) Cavitation response of SSPs, Dox or a mixture of the solutions in the CTR upon exposure to ultrasound for 10 minutes. “Cavitation signal” is the root mean square (in Volts). (**B**) The fluorescence of a serial dilution of insonated Dox and SSPs in comparison with a positive control of non-ultrasound-treated Dox and a negative control of Dox denatured by heating and hydrogen peroxide exposure. (**C**) The viability of A549 cells after incubation with 4 μM Dox, or glucose and SSP samples diluted an equivalent amount measured using an MTS assay. Data represent the mean of N=3, and standard deviation is shown. ANOVA demonstrated no significant difference between any groups containing Dox. **Abbreviations:** ANOVA, analysis of variance; Dox, doxorubicin; SSPs, sonosensitive particles; CTR, cavitation test rig; US, ultrasound; MTS, [3-(4,5-dimethylthiazol-2-yl)-5-(3-carboxymethoxyphenyl)-2-(4-sulfophenyl)-2H-tetrazolium.

**Figure 2:**
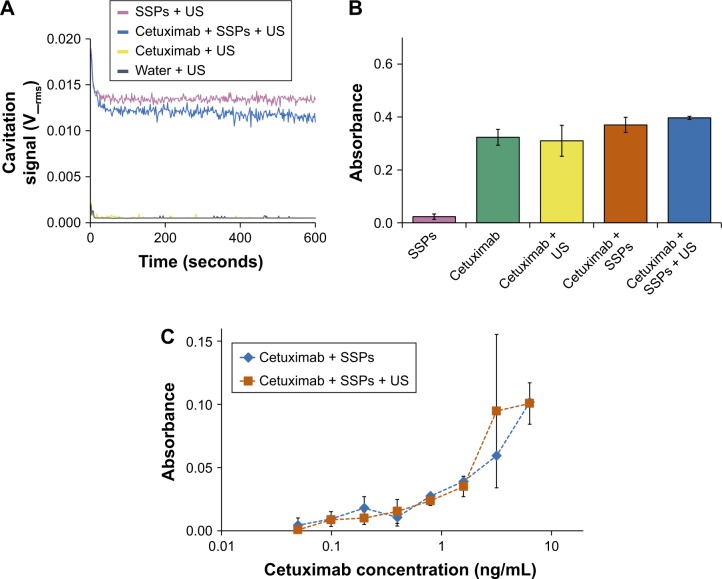
Assessment of whether cavitation alters the binding affinity of cetuximab. **Notes:** (**A**) Cavitation response of SSPs, cetuximab, or a mixture of the solutions in the CTR. “Cavitation signal” is the root mean square (in Volts). A bridging ELISA to assess alteration to the binding capacity of cetuximab to its target, epidermal growth factor receptor, or the structure of the cetuximab using an HRP-labeled anti-cetuximab antibody where absorbance is directly correlated with binding in the ELISA. (**B**) ELISA absorbance of cetuximab at a concentration of 12.7 ng/mL compared to an equivalent concentration of control groups. (**C**) Absorbance as a function of cetuximab concentration for cetuximab + SSPs compared to an equivalent concentration range of cetuximab + SSPs + US of control groups. Data represent the mean of N=3, and standard deviation is shown. ANOVA demonstrated no significant difference between any groups containing cetuximab. **Abbreviations:** ANOVA, analysis of variance; SSPs, sonosensitive particles; CTR, cavitation test rig; ELISA, enzyme-linked immunosorbent assay; US, ultrasound; HRP, horseradish peroxidase.

**Figure 3:**
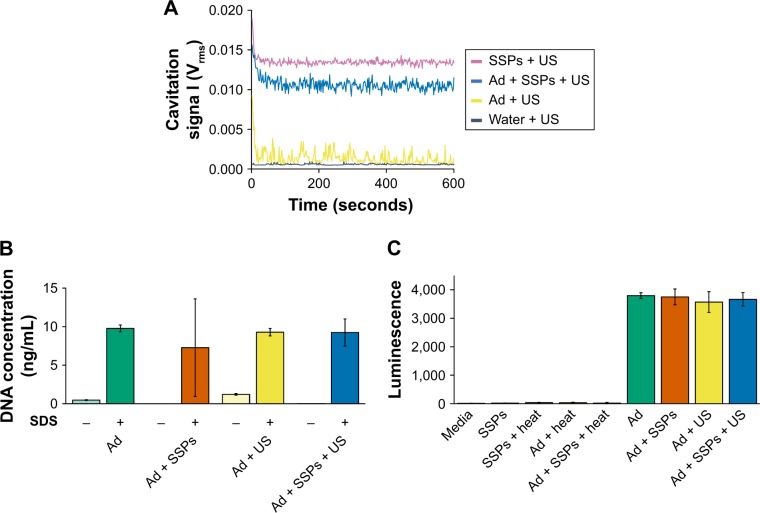
Impact of cavitation on the structural integrity and infectivity of Ad. **Notes:** (**A**) Cavitation response of Ad, SSPs or a mixture of the solutions in the CTR. “Cavitation signal” is the root mean square (in Volts). (**B**) Picogreen assay to investigate capsid stability to cavitation exposure. Hatched bars (denoted as “−”) represent samples not exposed to SDS and heat treatment, and filled bars (denoted as “+”) represent samples deliberately subjected to capsid disruption using SDS and heat treatment. (**C**) Luminescence in A549 cells incubated with Ad expressing a luciferase transgene 24 hours after exposure of the Ad to buffer, SSPs, US or SSPs and US. Data represent the mean of N=3, and standard deviation is shown. ANOVA demonstrated no significant difference between any groups containing Ad. **Abbreviations:** Ad, adenovirus; ANOVA, analysis of variance; SSPs, sonosensitive particles; CTR, cavitation test rig; SDS, sodium dodecyl sulfate; US, ultrasound.

**Figure 4:**
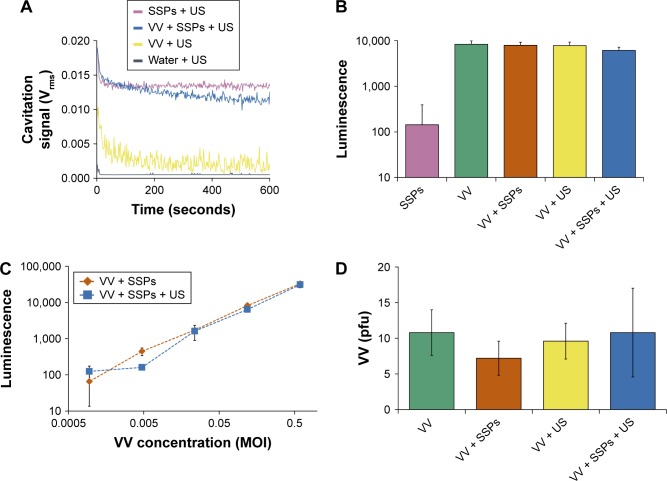
Impact of cavitation on infectivity of VV in cells incubated with the virus. **Notes:** (**A**) Cavitation response of VV, SSPs or a mixture of the solutions in the CTR. “Cavitation signal” is the root mean square (in Volts). Following exposure in the CTR, a luciferase-expressing version of the VV was incubated with A549 cells for 24 hours before cells were processed, luciferin was added and luminescence measured. (**B**) Luminescence of cells incubated with 0.12 MOI VV or control treatments at an equivalent dilution, N=3. (**C**) Luminescence as a function of VV concentration, N=3. (**D**) As an alternative measure of infection and spread, A549 cells were infected with a non-luciferase-expressing oncolytic VV, and the number of plaques counted 3 days later. Data represent the mean of N=5. Standard deviation is shown. **Abbreviations:** VV, vaccinia virus; SSPs, sonosensitive particles; CTR, cavitation test rig; MOI, multiplicity of infection; US, ultrasound; pfu, plaque-forming units.

To ensure tightly controlled, reproducible exposure to US-mediated cavitation events, an exposure chamber was designed and built (Figure S1). This cavitation test rig (CTR) comprised 2 US transducers (1 to expose and 1 to detect cavitation) aligned onto a 3 mL sealable cuvette (Sarstedt, code 67.754) containing sample. Samples were insonated within this cuvette in the CTR by a 4,000-cycle, 0.5 MHz burst, with a peak rarefactional pressure of 1.5 MPa and a pulse repetition frequency of 0.5 Hz generated by a submerged 25.4 mm diameter, 500 kHz unfocused transducer (Sonic Concepts, Bothell, WA, USA) positioned so it was 40 mm from the center of the exposure cuvette (parameters designed to match those used in in vitro and preclinical studies).^17^ As illustrated in Figure S1, the cavitation response was measured by a spherically focused broadband transducer (Fc =7.5 MHz, focus =73 mm; Olympus NDT, Waltham, MA, USA) positioned at 90° to the beam of the 500 kHz transducer so that the 2 foci overlap in the center of the sample. At each sonication, the focused transducer received acoustic emissions that were subsequently high pass filtered using an analog filter (cut-off frequency =5 MHz; Allen Avionics, Mineola, NY, USA) and 5 times amplified by a broadband preamplifier (Stanford Research Systems, Sunnyvale, CA, USA), as previously described.^18^ Finally, each of the received signals was digitized at 40 MHz (Pico Technology, Eaton Socon, UK) for analysis. The cavitation data are represented as a “cavitation signal” against time as shown in Figure S2 and Figures 1–4. The value of this signal at each time point is the root-mean-square value (in Volts) of the cavitation emission received on the receive transducer for that pulse. Each sample run was preceded and followed by the measurement of reference samples.

### Dox characterization

Samples were exposed to cavitation by placing them in the CTR for 10 minutes, after which they were recovered for analysis of structure and activity. To characterize whether the fluorescence profile of Dox was affected by cavitation, a serial 5-fold dilution of Dox samples was added to a 96-well plate, and the fluorescence at λ_ex_/λ_em_ 485/520 was measured. This was directly compared to the same dilution series of Dox that had either not been exposed in the CTR or had been heated to 50°C with hydrogen peroxide (Sigma; 216763) to act as negative and positive controls, respectively, for the unaffected or destroyed chemotherapy agent. Further analysis was performed using proton nuclear magnetic resonance (^1^H-NMR) spectroscopy. Samples were prepared as described above (1.27 mg/mL SSPs, 455 μM of Dox), but were dissolved in unbuffered water, without FBS. Samples (0.4 mL each) were centrifuged to remove SSPs (14,000 g, 10 minutes). The supernatants were then filtered through 0.2 μm pore-size nylon membrane syringe filters (Sigma, Z259942), freeze-dried and redissolved in 600 μL of DMSO-*d*_6_ (Sigma, 151874). ^1^H-NMR spectra were acquired on a Bruker Ascend 400 spectrometer with 30° pulses and a 6-second relaxation delay on a spectral width of 8,000 Hz. The spectra were apodized by multiplication with an exponential decay equivalent to 1.5 Hz line broadening and a Gaussian function equivalent to 1.5 Hz line broadening. The spectra were referenced to residual DMSO-*d*_5_. The structural assignment was made following Piorecka et al.^19^ The spectra produced pre- and post-cavitation exposure are shown in Figure S3.

To determine if the cytotoxicity of the Dox was impacted by cavitation, an MTS assay was performed. For this assay, 10,000 A549 cells (purchased from American Type Culture Collection, USA) in Dulbecco’s Modified Eagle Medium (DMEM; Thermo Fisher Scientific, 11965-092) containing 10% FBS were added per well in a clear 96-well plate and incubated overnight. A serial dilution of US- and non-US-treated Dox samples was added to the wells and incubated for a further 48 hours. Liquid in the wells was removed and replaced with MTS reagent (Promega, G3582), and media mixed at a ratio of 1:5. The absorbance of the wells was measured at 490 nm after approximately 30 minutes of incubation.

### Cetuximab characterization

The impact of cavitation on the targeting capacity of cetuximab was measured using an enzyme-linked immunosorbent assay (ELISA). For this, 100 μL of 0.3 μg/mL epidermal growth factor receptor (EGFR; Abcam, ab155639) in carbonate–bicarbonate buffer (Sigma, C3041) was added to each well of a maxisorb plate and incubated at room temperature for 2 hours. The plate was washed with phosphate-buffered saline (PBS; Fisher, 18912-014) containing 0.1% Tween-20 (Acros, 233362500) 3 times. Then, 100 μL of 10% FBS was added to the wells of the plate and incubated for 1 hour. This solution was replaced with 100 μL of the US-treated samples and control samples diluted in 10% FBS, and the plate was incubated for 1 hour. The plate was washed 3 times with PBS/Tween, and 100 μL of 1 μg/mL horseradish peroxidase (HRP)-labeled anti-cetuximab antibody (Bio-Rad, HCA228P) was then added to the plate and incubated for 1 hour. The plate was washed 5 times with the PBS/Tween solution before a final incubation with 100 μL 3,3′,5,5′-tetramethylbenzidine (TMB) substrate (SLS, 11484281001) for approximately 10 minutes. Then, 100 μL sulfuric acid (SLS, 72266) was added to the plate to stop the enzymatic reaction, and the absorbance of the wells at a wavelength of 450 nm was measured. Heating cetuximab to 100°C for 10 minutes provided validation that denaturation of the protein would lead to loss of function as detected by the ELISA (Figure S4). Sodium dodecyl sulfate polyacrylamide gel electrophoresis (SDS-PAGE) analysis was used to test the maintenance of cetuximab structure and molecular weight upon exposure to cavitation (Figure S5). Samples were prepared as described above (1.27 mg/mL SSPs, 126.9 μg/mL cetuximab), but were dissolved in water, without FBS. After exposure in the CTR, samples were diluted 3:1 in 4X Laemmli Sample Buffer (BioRad, 1610747) supplemented with 10% 2-mercaptoethanol (Sigma, M6250), and subsequently heated to 95°C for 10 minutes. Prior to dilution in sample buffer, a sample of untreated cetuximab was heated to 100°C for 10 minutes. Then, 10 μL of each sample was added per well to a 4%–20% precast polyacrylamide gel (BioRad, 4561096), and 10 μL of protein standard ladder (BioRad, 1610375) was added to the first lane for molecular weight determination. The gel was run in Tris–glycine–sodium dodecyl sulfate (SDS) running buffer (BioRad, 1610732) at a constant voltage of 160 V for 45 minutes. The gel was then fixed for 20 minutes (in a solution of 50% methanol [Fisher, 10499560], 10% acetic acid [Sigma, 320099] and 40% water), stained for 2 hours (in a solution of 0.05 g Coomassie Brilliant Blue R Dye [Sigma, B7920], 45 mL methanol, 10 mL acetic acid and 45 mL water) and finally destained overnight (in a solution of 50% methanol, 10% acetic acid and 40% water).

### Virus characterization

A DNA fluorescence assay (Quant-iT™ PicoGreen^®^ dsDNA Assay Kit; Thermo Fisher Scientific P7589) was used to assess the structural integrity of the Ad capsid after insonation. Ability of the dye to penetrate through the protein capsid into the core of the Ad where its DNA genome resides and then intercalate, thereby causing fluorescence emission, is directly related to the structural integrity of the capsid. Half of the US- and non-US-treated Ad samples were diluted in 10 mM Tris–HCl and 1 mM ethylenediaminetetraacetic acid (pH 7.5; 1X TE; Thermo Fisher Scientific, P7589), while the other half were diluted in 1X TE solution supplemented with 0.05% SDS (Fisher, 10182440). Serial dilutions of a Lambda DNA standard (Thermo Fisher Scientific, P7589) were prepared in the same solutions as the samples (1X TE plus or minus 0.05% SDS). The samples treated with SDS were also heated to 56°C for 10 minutes. In a 96-well plate, 50 μL of each sample and standard was mixed with 100 μL of Quant-iT™ PicoGreen^®^ dsDNA reagent (Thermo Fisher Scientific, P7589) diluted 1:200 in 1X TE and allowed to stand for 5 minutes before the fluorescence was measured at λ_ex_/λ_em_ of 480/520.

The infectivity of both Ad type 5 and VV was measured using viruses that had been genetically engineered to express luciferase. For this, 10,000 A549 cells were added to 96-well plates in DMEM containing 10% FBS and incubated overnight. Serial dilutions of US-treated and non-US-treated virions were added to the plates and incubated for 24 hours. Then, 100 μL of 150 μg/mL luciferin (Fisher, 15225733) was added to the plate infected with VV, and the luminescence immediately measured. Twenty-four hours postinfection with Ad, media was removed from each well, and cells were washed with 100 μL of PBS and were lysed using lysis buffer (Promega, E4030) and 1 freeze–thaw cycle. Then, 25 μL of cell lysate was added to a white opaque polystyrene 96-well plate. Luminescence per well was measured immediately after addition of 25 μL Luciferase Assay Substrate (Promega, E4030).

### Plaque assay

The ability of VV to replicate and spread was assessed using a plaque assay. For this, 500,000 A549 cells were added to each well of a 6-well plate in a solution of DMEM containing 10% FBS and incubated overnight. Stock VV was diluted 1 in 5.75×10^6^ in media, and 0.5 mL added to each of the wells. The cells were incubated with the virus for 3 hours before the media was replaced with 1% agar mixed with media that had been heated to 42°C. The plates were kept at room temperature for approximately 10 minutes while the agar set and returned to the incubator. Three days later, 0.5 mL of 1% (v/v) neutral red stain concentrate in media was added to each well, and well plates were returned to the incubator. The number of plaques was counted 3 hours later.

## Results

Cavitation is an intrinsically stochastic event, and so to ensure the levels of cavitation exposure were kept reproducible amongst replicates and between experiments a CTR was designed and constructed (Figures S1 and S2 show the schematic and validation of the CTR). By using a standardized US exposure regime of 10 minutes, 0.5 MHz, 1.5 MPa, 4,000 cycles, 0.5 Hz pulse repetition frequency, standard cavitation nuclei (SSPs), we could ensure cavitation levels were substantial, maintained over many cycles and could be compared to those shown to be successful in in vivo studies.^12^ SSPs have demonstrated more sustained cavitation than conventional US contrast agents such as SonoVue both in vitro and in vivo.^8^ Notably, analysis of the frequency spectra of the acoustic emissions recorded within the CTR shows a dominance of broadband signal in accordance with the propensity of the SSPs to produce inertial cavitation events^20^ (as evidenced by the broad range of frequencies detected; Figure S2B).

### Impact of cavitation on Dox

Having validated the CTR, it could then be used to reliably assess the impact of cavitation on drug structure and efficacy. As expected, in control groups lacking US or SSP cavitation nuclei, there was no cavitation signal detected (Figure 1A). In contrast when Dox was mixed with SSPs, and the standard US exposure regime applied, cavitation was detected between levels of 0.011 and 0.09 V_rms_ for the entire 10-minute duration. When Dox was assessed after exposure to cavitation, no alteration of its fluorescence at λ_ex_ 488 nm and λ_em_ 560 nm was observed (Figure 1B) compared to the control sample which was not exposed to cavitation; this indicates the multi-ring structure of Dox remained intact. In contrast, destruction of the Dox structure by oxidation (hydrogen peroxide and heating) ablated its fluorescence. These findings are in accordance with the studies of Lafond et al who demonstrated that cavitation instigated using peak negative pressures of 13 MPa and no cavitation nuclei did not impact on Dox structure, as shown by liquid chromatography and mass spectrometry.^21 1^H-NMR analysis was used to confirm our findings, with analysis demonstrating that Dox exposed to cavitation produced the same spectra as controls (Figure S3). To test for the maintenance of biological activity, samples were recovered from the CTR and added to a monolayer of A549 cells (Figure 1C). In all samples free of Dox, the cell viability was maintained at 100%, demonstrating SSPs or buffer alone to be nontoxic. In contrast, all Dox containing samples showed a reduction in cell viability to 5%–10% of that observed with the controls lacking Dox. ANOVA with comparison of all sample groups demonstrated no statistical difference between the cytotoxicity of any Dox-containing group (*P*>0.05). This indicates that the exposure of Dox to marked and sustained levels of cavitation (similar to those that have been shown to impact on drug delivery in vitro and in vivo) does not impact on the ability of the Dox to kill cells.

### Impact of cavitation on cetuximab

Protein-based therapeutics such as antibodies represent an increasingly important part of the clinical armamentarium,^22^ due to their high levels of specificity and decreased toxicity. However, as the efficiency of target binding depends on the maintenance of protein structural integrity, cavitation events have the potential to decrease therapeutic efficacy. To probe this, the activity of anticancer agent cetuximab (Erbitux) was tested following exposure in the CTR. Specifically, the capacity of cetuximab to bind its target, the EGFR, was assayed by ELISA. As in Figure 1A, only in the presence of US and SSPs within the CTR was a consistent level of cavitation maintained over the 10-minute duration (Figure 2A). When samples were tested for EGFR target binding by ELISA, SSPs alone gave no signal above background whereas strong binding to the ELISA plate was observed for all samples containing cetuximab (Figure 2B). Indeed, all these cetuximab-containing samples showed the same level of EGFR binding (*P*>0.05, 1-way ANOVA, Tukey’s test comparing all groups) regardless of the presence or absence of SSPs or whether they had been exposed to US or not. Heating of cetuximab to 100°C to ensure denaturation demonstrated that loss of structure would indeed lead to a loss of EGFR binding (Figure S4). When the maintenance of cetuximab molecular weight was tested using SDS-PAGE (Figure S5), there was no change in the intensity or migration distance of the heavy or light chains of the cavitation-exposed antibody compared to controls. Cetuximab denatured by heating (10 minutes at 100°C) as a positive control showed dramatic loss of band intensity for both heavy and light chain fragments.

When the cetuximab concentration dependence of the ELISA signal was probed (Figure 2C) over a range of 0.1–10 ng/mL, it was demonstrated that there was no difference (*P*>0.05) in EGFR binding between cetuximab + SSPs and cetuximab + SSPs + US samples at any concentration. The data provide strong evidence that exposure to cavitation does not impact detrimentally on the ability of antibody therapeutics to bind their biological targets.

### Impact of cavitation on non-enveloped virus

The use of selectively replicating viral vectors in the treatment of cancer represents an important development,^23^ but the potential of this approach may ultimately be stymied unless better delivery into and throughout solid tumors can be achieved.^24^,^25^ Cavitation provides a means to enhance delivery but also has the potential to damage the virus nucleotide, protein or lipid components. While some viral vectors are non-enveloped (Ad), others are dependent on the maintenance of their surrounding envelopes which comprise lipid layers with embedded proteins (VV).

In accordance with Figures 1A and 2A, the CTR was used to achieve marked and sustained exposure of Ad to cavitation (Figure 3A), with negligible detectable cavitation in the absence of SSPs but levels of between 0.01 and 0.015 V_rms_ for the entire 10-minute exposure when SSPs were present. The structural integrity of Ad can be assessed using the ability of Picogreen dye to enter its protein capsid and intercalate into its DNA genome (an event that increases fluorescence emission of the dye). Figure 3B demonstrates that exposure of Ad to sustained cavitation did not impact on the ability of its capsid to maintain integrity and exclude Picogreen. Notably, subsequent heating/SDS treatment resulted in capsid disruption and equivalently high levels of Picogreen fluorescence for all samples (*P*>0.05).

The impact of cavitation on the biological activity of Ad was probed by measuring the ability of Ad to infect a cancer cell line and express a luciferase reporter transgene (Figure 3C). It is apparent that samples exposed to cavitation gave the same levels of reporter gene expression (approximately 4,000 light units/well) as samples that were not exposed to cavitation (*P*>0.05). When destroyed by heating, the Ad failed to elicit expression of the luciferase transgene. Data in Figure S6 confirmed there was no difference in infectivity for samples exposed to, and not exposed to, cavitation across a range of Ad concentrations from 625 to 5,000 viruses per cell.

### Impact of cavitation on enveloped virus

Cavitation events have been shown to induce sonoporation events in which holes are created in cell plasma membranes.^26^ Cells have the capacity to repair and reseal these holes, meaning the process is not necessarily cytotoxic. In contrast, lipid-/protein-coated viruses do not have this repair capacity, and so it is important to assess the impact of cavitation upon therapeutically useful enveloped viruses such as VV. Figure 4A confirms the exposure of the VV + SSPs sample to marked and sustained cavitation of between 0.01 and 0.015 V_rms_ throughout the 10 minutes. Notably, the level of cavitation in VV + US controls was detectable above the background achieved with water + US. This was not the case for Dox or antibody samples but may be a consequence of the lower purity or particulate nature of VV preparations. Although outside the scope of this present study, this minor but intriguing finding may warrant further investigation as it has implications for the further combination of these technologies, as it may suggest these viruses could be “self-cavitation nucleating”. When reporter gene expression from the VV-containing samples was measured (Figure 4B), no differences were detected with all samples reaching levels of approximately 10,000 light units per well (*P*>0.05). When probed further over a 5-log dilution series, no difference between VV + SSPs and VV + SSPs + US was detected at any concentration (Figure 4C). When a control sample of VV denatured using heat treatment was tested for infectivity, no luciferase signal was detected (Figure S7). The ability of oncolytic viruses to selectively self-amplify within target cancer cells, achieve lytic release and then repeat this process is one of the aspects that has generated such clinical and commercial interest in their development.^23^ The ability of VV to maintain oncolytic potential after exposure to cavitation was therefore assessed using a plaque assay as described here. It was evident that all samples regardless of exposure to cavitation gave rise to the same number of plaques (approximately 10 plaques, *P*>0.05), 3 days after infection of an A549 cell monolayer (Figure 4D).

## Discussion

Application of localized cavitation is a promising approach to address the challenge of poor extravasation of drugs into and throughout tumors.^12^,^13^,^27^ However, cavitation has been shown to be associated with temperature increases, shock waves, shear stresses and damage to proteins and membranes.^15^ It is clear that control and monitoring of cavitation events has allowed their application for enhanced tumor delivery of drugs without causing damage to cells or tissue,^5^,^12^ but information on the impact of cavitation on the drugs themselves is scarce. A study by Lafond et al has reported the impact of unseeded cavitation on Dox,^21^ but cavitation effects on newer therapeutics remain to be defined. This is of particular relevance as cancer drugs are becoming increasingly complex and prone to loss of activity due to loss of structure.^22^ Consequently, the impact of cavitation on the range of cancer therapeutics in clinical use and development needs to be assessed.

The CTR developed and described here provided a controlled and standardized environment to gauge the impact of cavitation on a range of therapeutic agents. In the presence of SSP cavitation nuclei,^8^ cavitation could be instigated and detected in samples for a period of 10 minutes. During this time, in all samples including water alone, there was a sharp initial decrease in the cavitation level detected, perhaps indicating the cavitation and destruction of air bubbles entrapped in the cuvette during sample loading.

Under certain exposure conditions, the instigation of inertial cavitation can result in raised temperature. Crucially, when temperature was monitored during exposure to the US parameters described here, no increase was detected, a finding which has also been reproduced with these parameters in vivo (data not shown). This is probably because, rather than a continuous-wave exposure, a low duty cycle is used in our set-up. This means the US is only “on” for approximately 2% of the time allowing dissipation of any heat generated.

Notably, neither the presence of the SSP cavitation nuclei nor the cavitation events that they mediated upon exposure to US led to a measurable impact on the activity of a small-molecule chemotherapeutic (Dox), an antibody therapeutic (cetuximab), a non-enveloped virus (Ad) or an enveloped virus (VV). Researchers of sonodynamic therapy have extensively investigated the potentially beneficial activating impact of cavitation events on a specific class of small-molecule agents.^28^ In studying the impact of cavitation on the activity of a wide range of drugs in a standardized set-up, we have addressed an under-researched area. However, the utility of US in food manufacturing and processing applications has also been explored, and so its impact on the structure of whey protein concentrate has been investigated.^29^ These studies used dramatically different exposure parameters (20 kHz for up to 60 minutes), but even under these more extreme and potentially damaging regimes only minor changes to whey protein structure was observed. The cavitation nuclei and the US parameters we have explored here were designed to match the conditions we have shown to provide effective delivery of therapeutic agents in preclinical studies without causing cavitation-mediated damage to target or nontarget tissues.^13^ This provides confidence that these data can be extrapolated and therefore provide evidence for the amenability of all the drug classes tested to cavitation-mediated delivery into tumors.

## Supplementary materials

Figure S1 Schematic of the CTR.
**Note:** Sample was loaded into a 3 mL cuvette, and the cuvette placed in the holder to align it precisely with the transducers.
**Abbreviation:** CTR, cavitation test rig.

Figure S2 Characterization of CTR reproducibility.
**Note:** (**A**) Sustained cavitation activity over 10 minutes of sonication for 12 samples of SSPs. “Cavitation signal” is the root mean square (in Volts). (**B**) Broadband spectrum of SSP cavitation in the CTR from N=5 samples.
**Abbreviations:** CTR, cavitation test rig; SSPs, sonosensitive particles; SE, standard error.

Figure S3 ^1^H-NMR spectra of doxorubicin hydrochloride before and after exposure to ultrasound in the presence or absence of SSPs.
**Note:** Samples (0.4 mL each) were centrifuged to remove SSPs (14,000× *g*, 10 minutes). The supernatants were then filtered through 0.2 μm pore-size nylon membrane syringe filters, freeze-dried and redissolved in 600 μL of DMSO-*d*_6_. The structural assignment was made following Piorecka et al.^1^
**Abbreviations:** SSPs, sonosensitive particles; Dox, doxorubicin; US, ultrasound.

Figure S4 Demonstration that the cetuximab ELISA will not detect denatured cetuximab.
**Notes:** The EGFR-binding ability of a serial dilution of cetuximab was compared to that of a dilution series of heat-treated (100°C, 10 minutes) cetuximab. Data represent the mean of N=3, and standard deviation is shown.
**Abbreviations:** ELISA, enzyme-linked immunosorbent assay; EGFR, epidermal growth factor receptor.

Figure S5 Impact of cavitation on the molecular weight of cetuximab analyzed by SDS-PAGE: (1) protein standard ladder; (2) untreated cetuximab; (3) cetuximab + SSPs; (4) cetuximab + US; (5) cetuximab + SSPs + US; and (6) heat-denatured cetuximab.
**Notes:** Samples 2–6 were diluted 3:1 in Laemmli sample buffer supplemented with 10% 2-mercaptoethanol, and heated to 95°C for 10 minutes. Sample 6 was pretreated by boiling at 100°C for 10 minutes prior to dilution in sample buffer. After boiling, 10 μL of sample (0.95 μg of antibody) was added per well into a 4%–20% polyacrylamide gel. The gel was run in Tris–glycine–SDS buffer at 160 V for 45 minutes.
**Abbreviations:** SSPs, sonosensitive particles; US, ultrasound; SDS, sodium dodecyl sulfate; MW, molecular weight; PAGE, polyacrylamide gel electrophoresis.

Figure S6 Luciferase expression in cells incubated with a serial dilution of insonated or non-insonated mixture of Ad and SSPs.
**Notes:** The trend between Ad concentration and transgene expression was no different between the Ad treatment groups. Data represent the mean of N=3, and standard deviation is shown.
**Abbreviations:** Ad, adenovirus; SSPs, sonosensitive particles; US, ultrasound; MOI, multiplicity of infection.

Figure S7 Demonstration that the luminescence of cells incubated with luciferase-expressing VV would not occur if the VV had been denatured.
**Notes:** A549 cells were incubated with a serial dilution of non-heated VV or heat-inactivated VV. Luciferin was added to the cells 24 hours later, and luminescence immediately measured. Data represent the mean of N=3, and standard deviation is shown.
**Abbreviations:** VV, vaccinia virus; MOI, multiplicity of infection.

Reference1PioreckaKStanczykWFlorczakMNMR analysis of antitumor drugs: doxorubicin, daunorubicin and their functionalized derivativesTetrahedron Lett2017582152155
