# Supplementary figures and images for: Ultrasound-mediated cavitation does not decrease the activity of small molecule, antibody or viral-based medicines
Source: Int J Nanomedicine. 2018 Jan 10;13:337–49. doi: 10.2147/IJN.S141557 (PMC5768183; doi:10.2147/IJN.S141557)

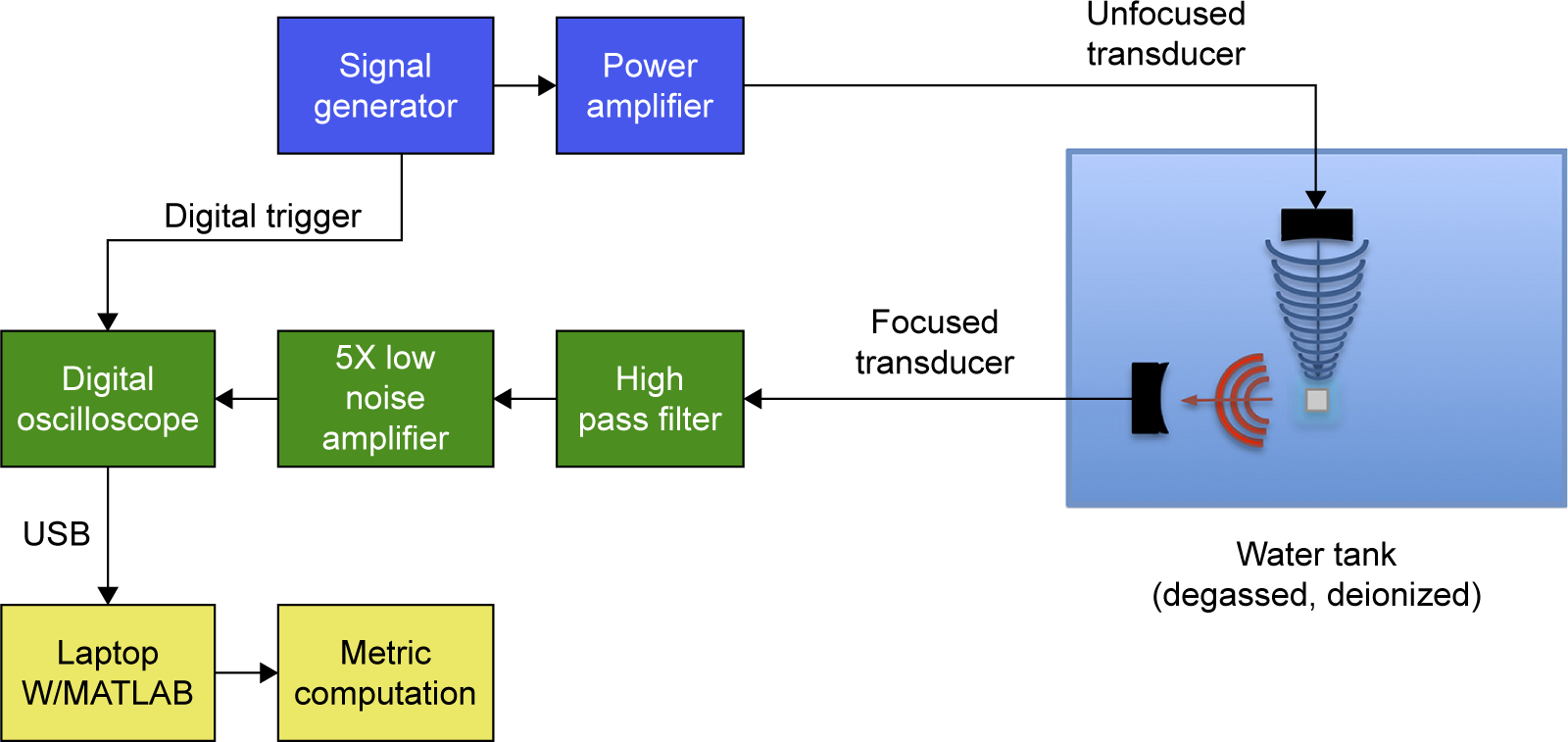

Supplement: Figure S1 — Schematic of the CTR. Note: Sample was loaded into a 3 mL cuvette, and the cuvette placed in the holder to align it precisely with the transducers. Abbreviation: CTR, cavitation test rig. [file ijn-13-337s1.tif]

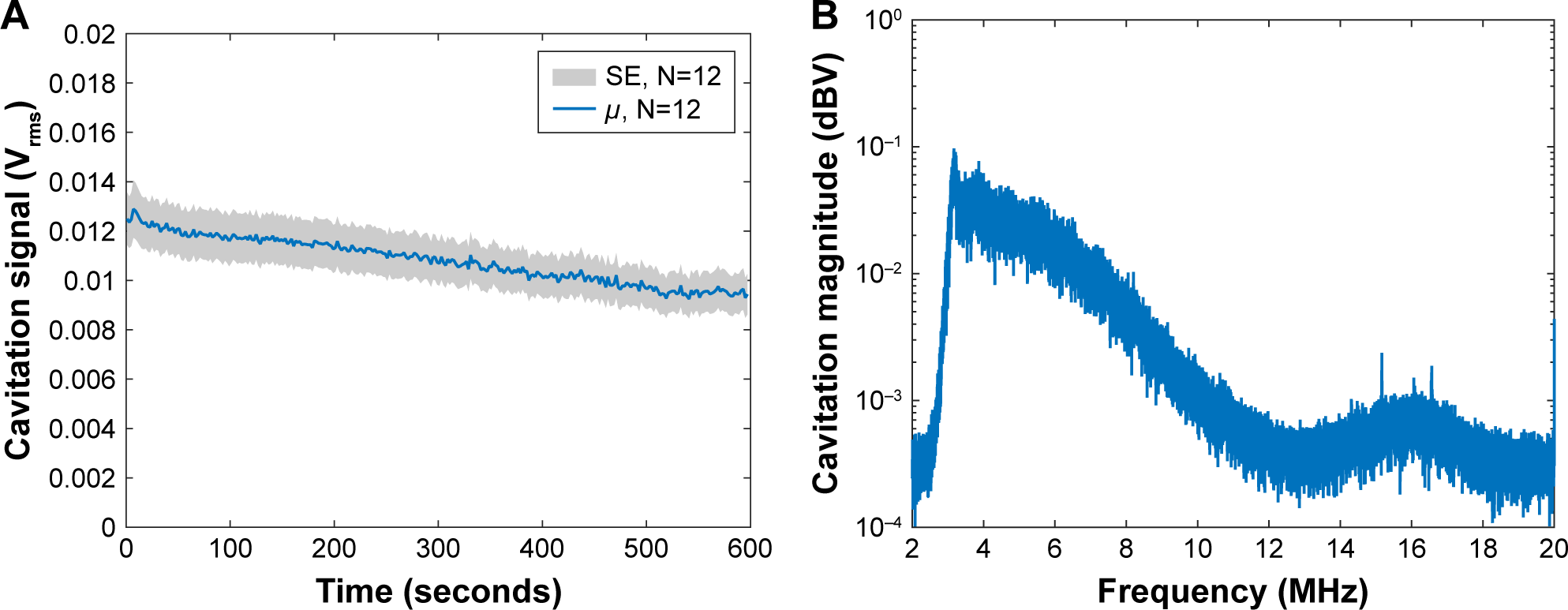

Supplement: Figure S2 — Characterization of CTR reproducibility. Note: (A) Sustained cavitation activity over 10 minutes of sonication for 12 samples of SSPs. “Cavitation signal” is the root mean square (in Volts). (B) Broadband spectrum of SSP cavitation in the CTR from N=5 samples. Abbreviations: CTR, cavitation test rig; SSPs, sonosensitive particles; SE, standard error. [file ijn-13-337s2.tif]

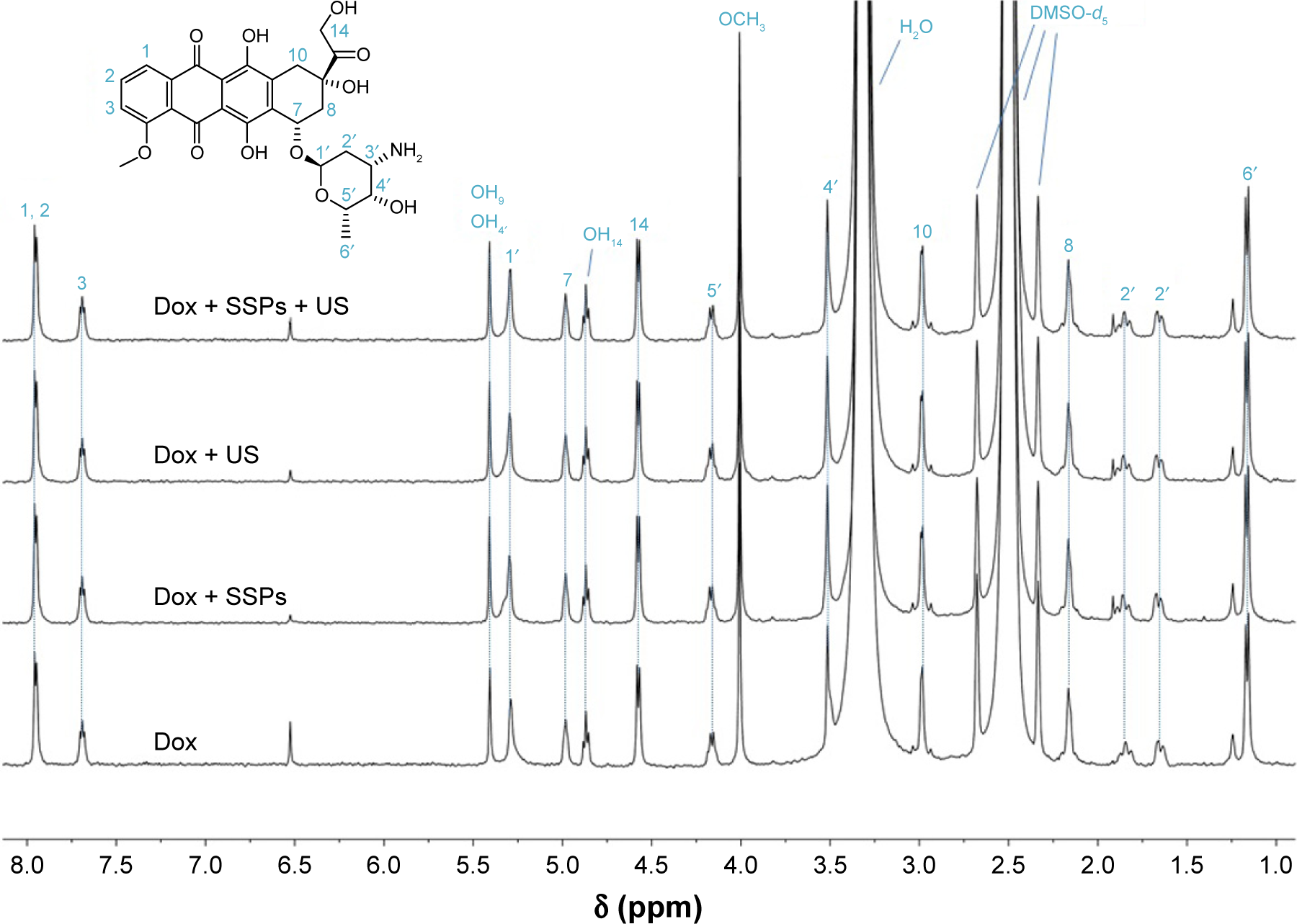

Supplement: Figure S3 — 1H-NMR spectra of doxorubicin hydrochloride before and after exposure to ultrasound in the presence or absence of SSPs. Note: Samples (0.4 mL each) were centrifuged to remove SSPs (14,000× g, 10 minutes). The supernatants were then filtered through 0.2 μm pore-size nylon membrane syringe filters, freeze-dried and redissolved in 600 μL of DMSO-d6. The structural assignment was made following Piorecka et al.1 Abbreviations: SSPs, sonosensitive particles; Dox, doxorubicin; US, ultrasound. [file ijn-13-337s3.tif]

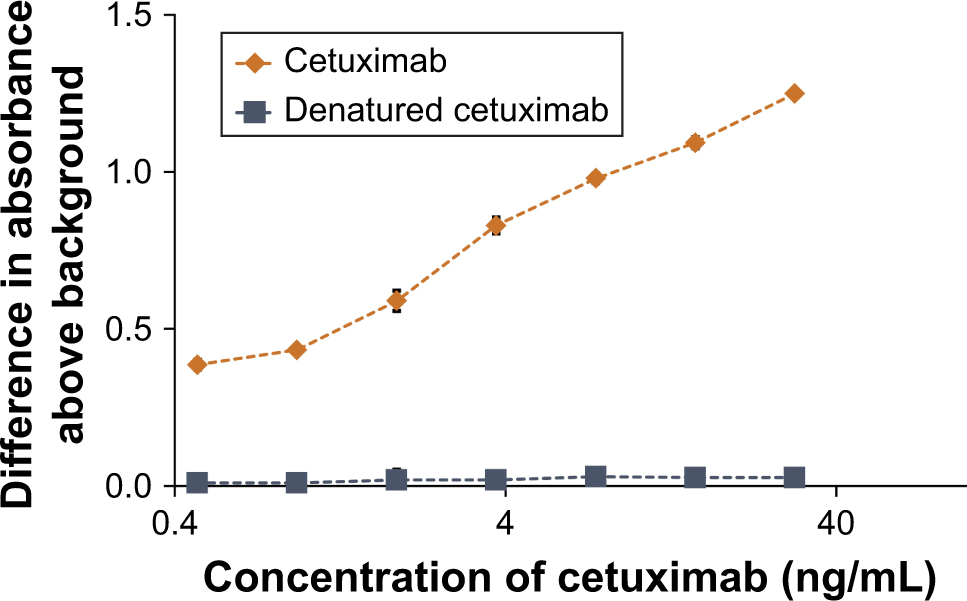

Supplement: Figure S4 — Demonstration that the cetuximab ELISA will not detect denatured cetuximab. Notes: The EGFR-binding ability of a serial dilution of cetuximab was compared to that of a dilution series of heat-treated (100°C, 10 minutes) cetuximab. Data represent the mean of N=3, and standard deviation is shown. Abbreviations: ELISA, enzyme-linked immunosorbent assay; EGFR, epidermal growth factor receptor. [file ijn-13-337s4.tif]

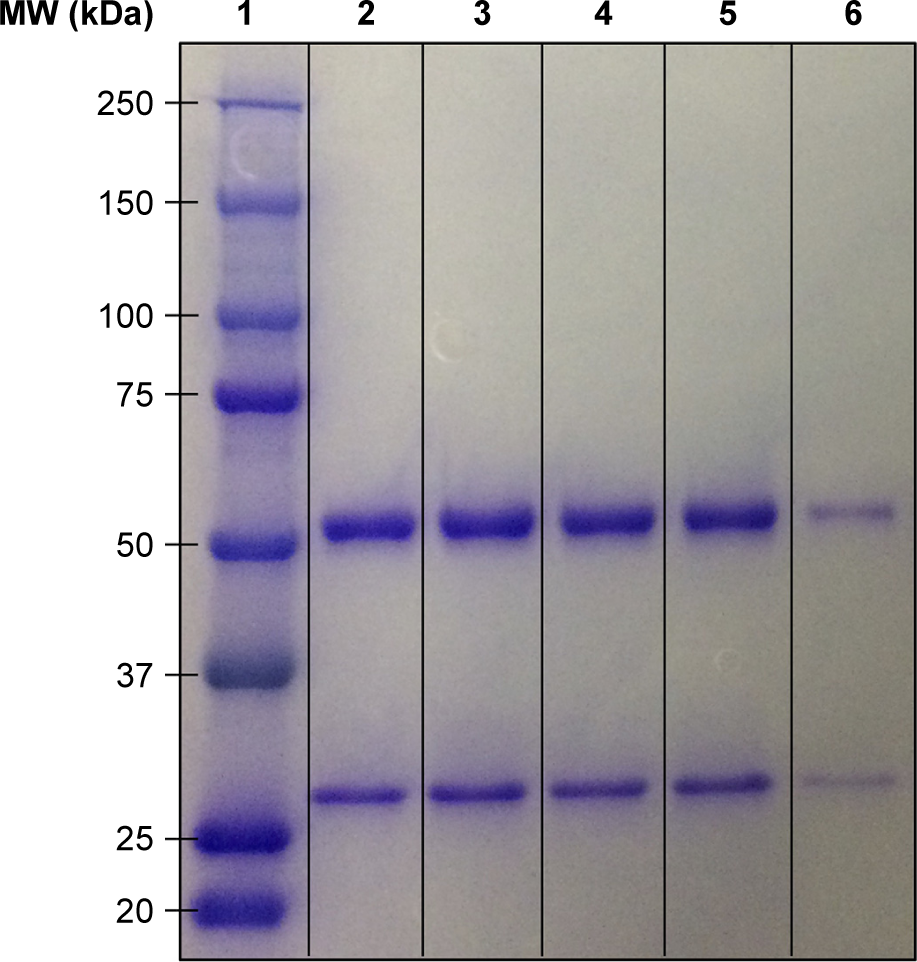

Supplement: Figure S5 — Impact of cavitation on the molecular weight of cetuximab analyzed by SDS-PAGE: (1) protein standard ladder; (2) untreated cetuximab; (3) cetuximab + SSPs; (4) cetuximab + US; (5) cetuximab + SSPs + US; and (6) heat-denatured cetuximab. Notes: Samples 2–6 were diluted 3:1 in Laemmli sample buffer supplemented with 10% 2-mercaptoethanol, and heated to 95°C for 10 minutes. Sample 6 was pretreated by boiling at 100°C for 10 minutes prior to dilution in sample buffer. After boiling, 10 μL of sample (0.95 μg of antibody) was added per well into a 4%–20% polyacrylamide gel. The gel was run in Tris–glycine–SDS buffer at 160 V for 45 minutes. Abbreviations: SSPs, sonosensitive particles; US, ultrasound; SDS, sodium dodecyl sulfate; MW, molecular weight; PAGE, polyacrylamide gel electrophoresis. [file ijn-13-337s5.tif]

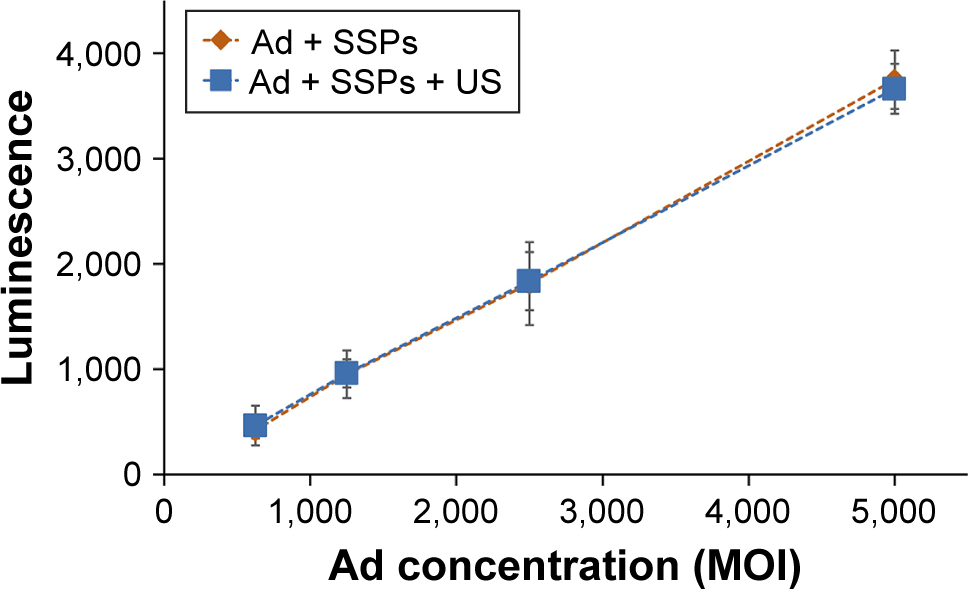

Supplement: Figure S6 — Luciferase expression in cells incubated with a serial dilution of insonated or non-insonated mixture of Ad and SSPs. Notes: The trend between Ad concentration and transgene expression was no different between the Ad treatment groups. Data represent the mean of N=3, and standard deviation is shown. Abbreviations: Ad, adenovirus; SSPs, sonosensitive particles; US, ultrasound; MOI, multiplicity of infection. [file ijn-13-337s6.tif]

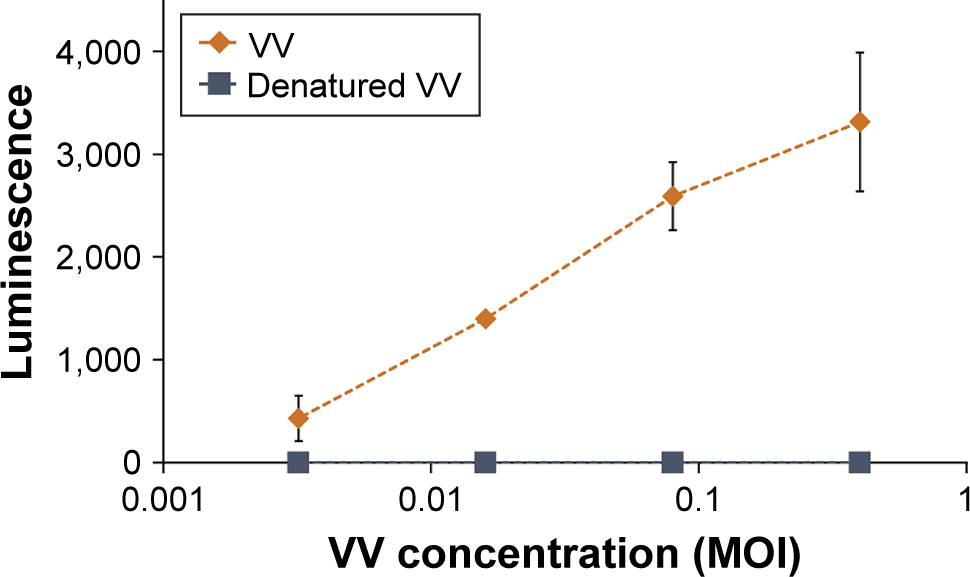

Supplement: Figure S7 — Demonstration that the luminescence of cells incubated with luciferase-expressing VV would not occur if the VV had been denatured. Notes: A549 cells were incubated with a serial dilution of non-heated VV or heat-inactivated VV. Luciferin was added to the cells 24 hours later, and luminescence immediately measured. Data represent the mean of N=3, and standard deviation is shown. Abbreviations: VV, vaccinia virus; MOI, multiplicity of infection. [file ijn-13-337s7.tif]
